# Autoimmune Diseases and Psychotic Disorders

**DOI:** 10.3389/fpsyt.2019.00131

**Published:** 2019-03-20

**Authors:** Rose Jeppesen, Michael Eriksen Benros

**Affiliations:** Mental Health Centre Copenhagen, Gentofte Hospital, Copenhagen University Hospital, Hellerup, Denmark

**Keywords:** autoimmune, immune system, psychosis, schizophrenia, mental illness

## Abstract

The notion of immunological pathways playing a role in the etiology of a subset of psychotic disorders has received increased interest in the last decades. One of the findings that has spiked interest herein, is an apparent link between autoimmune diseases and psychotic disorders. This is supported by genetic findings associating immune-related genetic markers with schizophrenia and clinical studies finding increased levels of inflammatory markers in patients with psychosis. Several large-scale epidemiologic studies have found positive associations between autoimmune diseases and psychosis. Particularly, autoimmune diseases as multiple sclerosis and lupus are known to have higher frequencies of neuropsychiatric symptoms, including psychosis, compared to healthy controls. Cross sectional studies have found higher prevalence of psychiatric diagnoses among those with autoimmune diseases, and longitudinal studies have shown bidirectional associations between several autoimmune diseases and increased risks associated with schizophrenia. Moreover, a family history of autoimmune diseases has been shown to be associated with an increased risk of psychotic disorders and vice versa. In this review we will summarize the epidemiologic evidence on associations between autoimmune diseases and psychosis. Possible mechanisms accountable for the association will be discussed, amongst others the probable role of shared genetic risk factors, the impact of infections on both autoimmunity and the development of psychotic disorders, and the potential role of the microbiome. We discuss the findings on and influence of autoantibodies and dysregulation of T- and B-cells in both disease categories, and why further research hereon is needed. In addition to the potential importance of autoimmunity in etiological mechanisms of psychotic disorders, the association also brings important attention to somatic comorbidity in patients with psychotic disorders.

## Introduction

The association between immunological processes and mental disorders was observed by doctors centuries before the immune system was discovered. Psychosis arising either with the occurrence or disappearance of acute fever has been described by many scientists from Hippocrates around 400 BC to Kraepelin around 1900. In the 1930s it was first hypothesized by Hermann Lehmann-Facius that schizophrenia was the product of an autoimmune reaction with antibodies attacking brain tissue (1). In the 1950s and 1960s it was noticed that celiac disease seemed to occur more often within those suffering from schizophrenia than in the general population (2), and conversely, that schizophrenia occurred less frequently within patients with rheumatoid arthritis (3, 4). Additionally, autoantibodies cross-reacting with brain antigens were found in patients with schizophrenia back in the 1960s (5, 6), and interest in anti-neuronal antibodies in psychotic disorders has increased during the last couple of decades, with an increasing number of reports on previously unknown antibodies with brain reactivity in patients suffering from psychosis (7–9).

The amount of evidence supporting the notion of a link between immunological processes and psychotic disorders has increased. Elevated levels of inflammatory markers have been found both in the blood (10, 11) and CSF (12–14) of patients with psychosis, with even higher levels in patients in first episode psychosis or acute relapse. Furthermore, some have found association between higher levels of inflammation in childhood and adolescence and increased risk of psychotic disorders (15, 16), elevated inflammatory biomarkers has been associated with lack of treatment response (17), and anti-inflammatory treatment has been found to have especially beneficial effect in an inflamed subgroup of patients (18–21). Moreover, it has been suggested that schizophrenia could be an autoimmune disease, based on similarities such as the remitting-relapsing phenotype of the illness, as well as the above-mentioned immunological processes (22).

Research in the field of psychoneuroimmunology is still evolving, with many different aspects being investigated. The notion of a role of the immune system in psychotic disorders seems evident, and understanding the link between autoimmune diseases and mental disorders may shed light on possible etiological mechanisms herein. Understanding how the immune system and psychotic illness interact can improve our understanding of psychosis and give rise to a wide range of new treatment options in psychiatry; amongst other the possibility to identify subgroups of patients with psychotic disorders and ongoing inflammatory processes that could benefit from more targeted treatment. Additionally, it is very important for clinicians to be aware of somatic comorbidities, particularly in patients with psychotic disorders, in order to improve detection and treatment, and thus the course of illness.

## Epidemiological Associations

The world-wide prevalence of schizophrenia is known to be around 1% (23) and the prevalence of autoimmune diseases have been found in a Danish nationwide study to be 4% (24). The vast majority of epidemiological studies have found a general association between autoimmunity and psychotic disorders (24–29). In large-scale register-based studies from Denmark, 6% of those diagnosed with schizophrenia also had a hospital contact related to an autoimmune disease during follow-up (25, 26), and a Taiwanese study found that 3.4% of persons with a hospital contact for autoimmune diseases also had a hospital contact related to schizophrenia (29). A Danish study based on 7704 patients with schizophrenia, found an increased prevalence by about 45% of the occurrence of an autoimmune disease (28), which was later confirmed in a Taiwanese population-based study (27). Regarding the risk of psychosis after an autoimmune disease diagnosis, a Danish nationwide study found this to be increased by 45%, which diminished to a 29% increased risk when excluding the effect of infections (26), and a very recent meta-analysis by Cullen et al. (30) found that a diagnosis of a non-neurological autoimmune disease increased the risk of later being diagnosed with a psychotic disorder by 43%.

Additionally, being diagnosed with schizophrenia increases the lifetime prevalence of autoimmune diseases. Two Danish register-based studies found that individuals with psychotic disorders had a subsequently elevated risk for autoimmune diseases by around 50% (25, 28). Supporting this, the recent meta-analysis found that the risk of having an autoimmune disease was 55% higher among those with a prior diagnosis of a psychotic disorder (30).

Autoimmune diseases and psychosis are not only associated on an individual level. Having a first degree relative with schizophrenia has also been found to increase the risk of autoimmune diseases with 6% (25), and a family history of autoimmunity has been found to increase risk of both schizophrenia and non-affective psychoses with 10% (24).

The associations with psychotic disorders have been found for a broad range of autoimmune diseases. For an overview of the associations between specific autoimmune diseases with psychotic disorders, please see Table 1 and the below sections.

**Table 1:** Associations found between autoimmune diseases and psychotic disorders.

**Autoimmune disorders**	**Studies with positive association**	**Positive ass. only with concurrent infection**	**Studies with no significant association**	**Studies with negative association**
Celiac disease	Chen et al (29), Cullen et al. (30), Benros et al. (26)		Benros et al. (25)	
Multiple sclerosis	Benros et al. (25), Benros et al. (26)		Wang et al. (27), Eaton et al. (28), Eaton et al. (24)	Johansson et al. (31)
Lupus	Wang et al. (27)	Benros et al. (26)	Cullen et al. (30), Benros et al. (25), Chen et al. (29), Eaton et al. (28), Eaton et al. (24)	
Graves/thyrotoxicosis	Chen et al. (29), Cullen et al. (30), Eaton et al. (28), Eaton et al. (24), Benros et al. (26)		Benros et al. (25)	
Autoimmune thyroiditis		Benros et al. (26)	Eaton et al. (28), Benros et al. (25)	
Diabetes type 1	Benros et al. (25), Eaton et al. (24), Benros et al. (26)		Chen et al. (29), Cullen et al. (30), Cremaschi et al. (32)	Juvonen et al. (33)
Rheumatoid arthritis	Wang et al. (27)	Benros et al. (26)	Eaton et al. (28), Eaton et al. (24)	Benros et al. (25), Chen et al. (29), Cullen et al. (30)
Psoriasis	Benros et al. (25), Chen et al. (29), Cullen et al. (30), Eaton et al. (24), Benros et al. (26)		Eaton et al. (28),	
Guillain-Barre	Benros et al. (25)	Benros et al. (26)	Eaton et al. (28),	
Crohns disease	Benros et al. (25)	Benros et al. (26)	Wang et al. (27), Cullen et al. (30), Eaton et al. (24)	
Autoimmune hepatitis	Benros et al. (25), Eaton et al. (28), Eaton et al. (24), Benros et al. (26)			
Pernicious anemia	Benros et al. (25), Cullen et al. (30), Chen et al. (29)			
Primary adrenocortical insufficiency	Benros et al. (25),			
Primary biliary cirrhosis	Benros et al. (25),			
Ankylosing spondylitis	Eaton et al. (24)	Benros et al. (26)	Benros et al. (25), Chen et al. (29), Eaton et al. (28)	Cullen et al. (30)
Sjögren syndrome	Eaton et al. (28)	Benros et al. (26)	Benros et al. (25), Chen et al. (29)	
Hypersensitivity vasculitis	Chen et al. (29)			
Haemolytic anemia	Eaton et al. (28)		Chen et al. (29)	
Pemphigoid	Cullen et al. (30)		Eaton et al. (28)	
Alopecia areata	Eaton et al. (28)		Benros et al. (25), Cullen et al. (30), Chen et al. (29)	
Polymylagia rheumatic	Eaton et al. (28)		Benros et al. (25), Chen et al. (29)	

### Celiac Disease

The original findings from the 1950s of an association between celiac disease and schizophrenia has since been explored further. During the next decades it was noticed that populations with lower consumption of wheat had lower incidence rates of schizophrenia (34–36), and small studies have since found beneficial effect on psychotic symptoms of a gluten-free diet in patients suffering from both celiac disease and schizophrenia (37, 38). One epidemiological study found no significant correlation between celiac disease and psychosis (24). However, another large-scale study found a 2.11-times increased risk of schizophrenia (26) and the recent meta-analysis also found an association with an elevated risk of schizophrenia with 53% (30). Additionally, it has been found in a Taiwanese population, that the risk of celiac disease is increased when suffering from schizophrenia (29). When discussing epidemiological studies using health records, it is important to note that celiac disease might be majorly underdiagnosed particularly within those who have already debuted with psychotic symptoms. In summary, most studies found a positive association between celiac disease and psychotic disorders.

### Multiple Sclerosis

Multiple sclerosis (MS) is an autoimmune disease associated with many neuropsychiatric symptoms, such as depression and anxiety (39). It has been found that 4% of patients with MS experiences psychosis (40), a prevalence much higher than that of the general population. Danish register-based studies have found that having MS increases the risk of schizophrenia with up to 44% (24, 26), with an even further increase in risk when having both MS and a prior hospital contact due to infection (26). Two studies found increased risk of schizophrenia with up to 30% in individuals with a family history of MS; however, they found no associations on an individual level (24, 28), and a study from Taiwan only found a trend toward an increased risk of schizophrenia in those with a diagnosis of MS (27). On the risk of a subsequent MS diagnosis in patients with schizophrenia, contradictory results have been found between a Danish and a Swedish nationwide study, finding the risk to be respectively increased by 57% (25) and decreased by 40% (31). Current evidence of an association between MS and psychotic disorders is limited with studies showing conflicting results. Many, especially sensory, symptoms of multiple sclerosis might be misinterpreted as part of the patients' psychotic disorders, complicating the diagnostic process, and psychotic symptoms in people with MS might not be diagnosed since they are considered to be delirium in relation to acute MS exacerbations.

### Lupus

Systemic Lupus Erythematosus (SLE) is another autoimmune disease known to have a high degree of neuropsychiatric problems, such as depression and anxiety, occurring in between 21 and 95% of patients (41). However, it has been estimated that only 13–38%, are directly attributable to SLE, whereas the remaining is suggested to be due to for example treatment complications (41). Regarding psychosis in SLE, the prevalence ranges from 2.3 to 11% in studies (42–44). A study from England comprising 458 patients with SLE, found that only 2.3% experienced psychosis (42), while a higher prevalence of psychosis have been found in a black Caribbean study population (366 patients, 7% with psychosis) (43) and in a Brazilian population (520 patients, 11% with psychosis) (44). In those experiencing psychosis, this was one of the initial symptoms of SLE in up to 60% of these patients (42). In population-based studies, a nationwide Taiwanese study found an increased risk of schizophrenia among those with SLE (27), and in one Danish study the presence of both SLE and a prior hospital contact with infection resulted in an increased risk of schizophrenia (26). None of the other epidemiological studies have found significant association between psychotic disorders and SLE (24, 25, 28, 30), but noteworthy, the number of cases available was very small in all studies, limiting the significance of possible findings. In summary, large scale studies with a greater number of cases have been able to find positive associations between SLE and psychotic disorders, while smaller studies have failed to do so.

### Autoimmune Thyroid Disorders

Graves' disease, the most common cause of hyperthyroidism, is also known to be linked to neuropsychiatric issues, and some even present with psychotic disorders (45). A German study found that in a cohort of 100 patients with a schizophreniform illness, 19 had increases antithyroid autoantibodies in sera, and 13 showed signs of intrathecal synthesis hereof (46). In epidemiological studies, both Graves' disease and thyrotoxicosis have been linked with an increased risk of schizophrenia (24, 26, 30). Additionally, the prevalence hereof has been found to be increased among individuals with schizophrenia (28, 29), though this finding has not been replicated in all studies (25, 32). Hence, most studies indicate a positive association between Graves' disease/thyrotoxicosis and psychotic disorders.

No studies has been able to show a significant association between autoimmune thyroiditis and schizophrenia on an individual level (24, 28), but one Danish study found an increased incidence among parents and siblings of patients with schizophrenia (28).

### Diabetes Type 1

Diabetes mellitus type 1 is a disease characterized by the presence of glutamic acid decarboxylase (GAD) antibodies. These autoantibodies have been linked with neurological problems (47), and thus have shown ability to cross the blood brain barrier, making them an interesting topic in the discussion of pathophysiological mechanisms. However, conflicting results have been found regarding the association of type 1 diabetes and psychotic disorders. Two Danish studies found an increased risk of schizophrenia when suffering from type 1 diabetes (24, 26), and one found an increased risk of type 1 diabetes after having been diagnosed with schizophrenia (25). This, however, was not replicated neither in a Swedish cohort (32), a Taiwanese cohort (29) nor in the recent meta-analysis (30), and a Finnish study even found a negative association (33). In summary, there does not seem to be a clear association between type 1 Diabetes and psychosis.

### Rheumatoid Arthritis

A disease which has consistently been found to be negatively associated with schizophrenia is rheumatoid arthritis (RA). This apparent “protective” effect of schizophrenia on the development of rheumatoid arthritis was investigated as early as the 1950s (48, 49). The negative association between the two has since been backed by epidemiological studies, finding decreased risk of schizophrenia in those with RA (30) and vice versa (25, 29, 50, 51). However, some studies did not find associations (24, 52), and regarding the association on the risk of psychosis after a RA diagnosis, more controversy exist, with a Danish study finding that a combined history of a hospital contact due to infection and RA increased the risk of schizophrenia (26) and a new Taiwanese study finding an increased risk of developing schizophrenia in individuals with a history of RA (27). Moreover, a Danish study found an increased prevalence of RA in the family of those with schizophrenia (28). One explanation of the consistent finding of negative association with subsequent RA diagnosis after a schizophrenia diagnosis could be that RA tends to be underdiagnosed in those suffering from psychotic disorder, and in concordance with this, both a Swedish and Danish nationwide study has shown that the same negative association can be found with other musculoskeletal diseases (50, 51).

### Autoimmune Encephalitis

Something that really spiked the interest in autoimmunity as a player in mental illness, was the discovery of autoimmune encephalitis. As a group, these diseases are characterized by the presence of neuronal surface antibodies (NSAbs) and symptoms include psychiatric and cognitive alterations, seizures and movement disorders, with the most commonly affected part of the brain being the limbic system. The most discussed antibody in psychotic disorders at the moment is the N-methyl-D-aspartate receptor (NMDA-R) antibody. It has been reported that as many as 74% of patients suffering from NMDA-R encephalitis experience psychotic symptoms (53, 54), and a recent smaller study found that 13% were initially admitted to the hospital with a psychiatric diagnosis (55). Multiple studies have investigated the frequency of NMDA-R antibodies in schizophrenia, but so far most have only had access to serum not CSF, most have had no healthy control group, and results have varied markedly (56).

### Other Autoimmune Diseases

Associations have been found between psychotic disorders and other autoimmune diseases as well. The incidence of psoriasis have been found to be significantly increased in individuals with schizophrenia (25, 29), but not in all studies (28). Increased incidence of psoriasis have also been found in individuals with a family history of schizophrenia (28). In addition, the risk of developing schizophrenia has been found in multiple studies to be increased in those with a history of psoriasis (24, 26, 30), with an additional increase when combined with a prior hospital contact due to an infection (26). The risk of developing Guillain-Barré syndrome, an autoimmune disease attacking peripheral nerves, has been found to be increased markedly in individuals with schizophrenia (25), and when having both a history of a hospital contact with an infection as wells as Guillain-Barré, the risk of developing schizophrenia has also been found to be increased (26). However, one other study found no association (28). Autoimmune hepatitis has been found to be greatly associated with psychotic disorders as well, with both individual history and family history hereof increasing the risk of schizophrenia (24, 26), and schizophrenia increasing the risk of autoimmune hepatitis (25). Some evidence of an association between schizophrenia and Crohn's disease has also been found (25, 26), though no significantly increased risk was shown in two other studies (24, 27) or the recent meta-analysis (30).

## Possible Mechanisms

**Figure 1:**
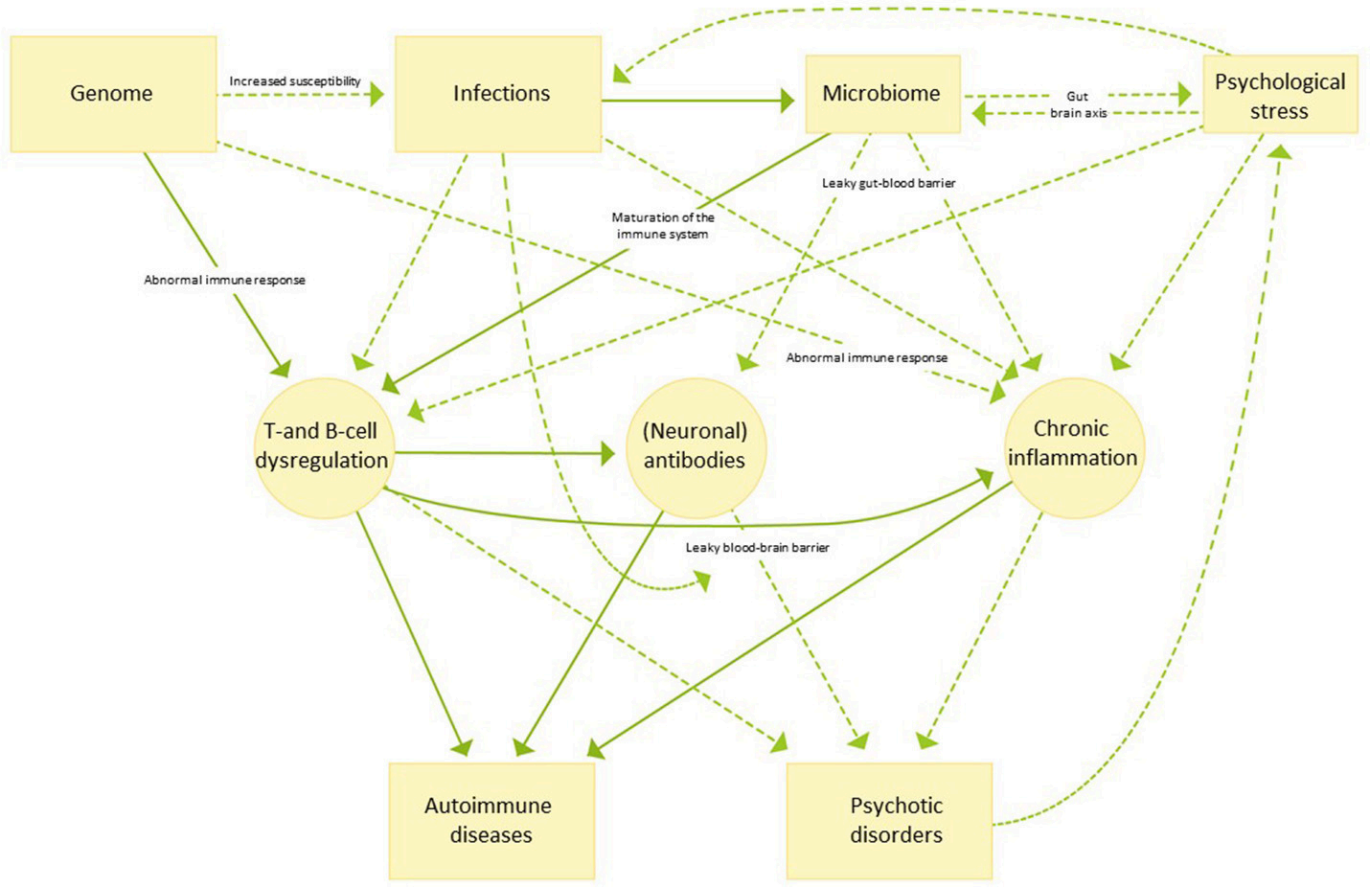
An illustrative overview of possible etiological mechanisms linking autoimmune disease and psychotic disorders. Continuous arrows indicate pathways for which evidence is strong, while dotted arrows indicate pathways which are currently not well understood or more speculative in nature.

The potential etiological background and the many factors that can influence the association between autoimmune diseases and psychosis are numerous and not mutually exclusive as outlined in the following sections. For an overview hereof, see Figure 1.

### Antibodies

One potential contributing factor to the link between some autoimmune diseases with mental illness, can be the presence of neuronal surface antibodies (NSAbs). GAD-antibodies have been linked with multiple neurological problems (47), and in neuropsychiatric lupus, an increased amount of antibodies was found both in serum and CSF compared to lupus with no neuropsychiatric manifestation (57). Furthermore, gliadin antibodies, associated with celiac disease, have been found to be increased in the serum of patients with recent onset schizophrenia (58). With the discovery of NMDA-receptor encephalitis, and its ability to mimic mental disorders, the interest spiked further, and with GAD-antibodies being able to induce limbic encephalitis (59, 60) and antibodies reacting with the NR2 subunit of NMDA being present in some cases of lupus (61), a possible link emerged.

Many studies have sought to evaluate the presence of multiple different NSAbs in mental illness, but so far, consistency in methods and assays have limited the generalization of the findings (56, 62). Many studies have lacked a healthy control group to compare their results with, and most studies have included serum but not CSF samples. The relevance of circulating NSAbs in serum is still unknown, and therefore comprehensive studies including healthy controls evaluating antibodies in both CSF and serum is needed to increase knowledge further.

### Dysregulated Immune System

A dysregulated balance between regulatory T cells and Th17 cells have been described to be essential for immunological homeostasis and have been implicated in the development of several autoimmune disorders (63). Signs of a dysregulated immune system has also been found in mental illnesses and might play a role in the association found between the two.

A meta-analysis found that levels of several lymphocytes differed when examining patients with schizophrenia compared to healthy controls (64), and studies have linked decreased regulatory T cells with negative symptoms and cognitive deficits (65), as well as increased levels of Th17 with psychopathology (66).

In recent years, B cells have received increasing attention in the pathology of autoimmunity, and have been implicated to play a big role in for example MS, where it has also been found that anti B-cell antigen (anti-CD20) have great efficacy in the treatment hereof (67). It has been shown that oligoclonal bands (OCBs) in the CSF, something which is found in approximately 90% of patients with MS, is a sign of ongoing stimulation and maturation of antibody-expressing B-cells (68). Interestingly, a recent meta-analysis found that OCBs were found in the CSF of up to 12.5% of patients with schizophrenia (14).

Another frequent finding in patients with schizophrenia is increased levels of pro-inflammatory and decreased levels of anti-inflammatory cytokines in serum (10). Dysregulation of the anti-inflammatory cytokine IL-10 has been found to be linked with abnormal responses to common infections, and to increase the risk of developing autoimmune diseases (69).

### Infections as a Common Risk Factor

It is thought that one of the most important triggers for developing autoimmune diseases is infection (70), and it is known that infectious encephalitis, specifically with herpes-simplex virus, markedly increases the risk of developing NMDA-receptor encephalitis (71). As it was shown in a large Danish nationwide study, prior infection increased the risk of developing schizophrenia in a dose-response fashion (26), and this finding has been repeated in other large studies (72, 73). The effect of infection on risk of schizophrenia was present regardless of autoimmune diseases, but additionally, a significant synergy was found in those with both a history of autoimmunity and infections (26). For many of the individual autoimmune diseases, it was seen that the effect on the risk of schizophrenia increased when a prior hospital contact due to infection was also present (26).

Being exposed to viral or bacterial infection is known to increase the permeability of the blood-brain-barrier (BBB) (74), which allows the entering into the central nervous system of immune cells and pro-inflammatory cytokines. This in itself might allow an inflammatory state in the brain, which has been theorized to play a role in the development of psychotic disorders. It may also explain the synergistic effect on risk of schizophrenia of having both an autoimmune disease and prior infections, as BBB disruption might also allow the entering of circulating antibodies. Supporting the role hereof, signs of a disrupted BBB has been found in patient with schizophrenia with evidence of increased albumin CSF:plasma ratio (75, 76) and increased levels of circulating s100-b (77).

It has also been found that infections during pregnancy increases the risk of schizophrenia in the offspring (78). On the basis hereof, it has been considered whether infections during the prenatal phase might prime the immune system, making it more vulnerable and perhaps more likely to produce abnormal responses to later infections, resulting in increased inflammation. However, a new study have shown that even maternal infections before and after pregnancy increases the risk of mental illness (79), which could also indicate a genetic susceptibility for infections associated with mental illness.

### Genetics

Both schizophrenia and autoimmune diseases are known to be highly hereditable. The most consistent finding in genetic studies of patients with schizophrenia, are differences in genes known to be linked to the immune system (80), and several genetic loci that increases the risk of autoimmune diseases has been located (81). As with schizophrenia, some of the discovered genetic loci in autoimmune diseases are located in the MHC region (82). However, while one study found significant overlap in genes between MS and schizophrenia (but not MS and bipolar disorder) (83), another study found no genetic association between 25 different autoimmune diseases and schizophrenia (84). Genetic pleiotropy has also been hypothesized to account for the negative association found between RA and schizophrenia, with genes found to be associated with schizophrenia possibly decreasing the risk of RA (85).

Another possible role of genetics in the association of autoimmune diseases and psychotic disorders could be a hereditary susceptibility for shared risk factors. It has been hypothesized that some of the genetic findings associated with schizophrenia might increase the risk of having infections (86, 87), that then subsequently increase the risk of both autoimmune diseases and psychotic disorders. Additionally, it has been theorized whether some individuals with schizophrenia, might have a genetic predisposition for an abnormal immune response to common infections and foreign pathogens, for example via differences in the HLA region and complement system (88, 89), which in turn could increase the risk of developing autoimmune reactions. The complement system has also been implicated to play a role in neurodevelopment and -maturation (90), and evidence of altered complement activity in patients with schizophrenia have been found (reviewed in (88)).

### The Microbiome

The gastro-intestinal tract of humans contains vast amounts of bacteria, phyla and other microorganisms, their genes collectively known as the microbiome, containing at least 100 times more genetic material than the human genome (91). This area has received great attention in the research of many different illnesses in the last years and have been implicated as a possible etiological factor in both neuropsychiatric illnesses and autoimmune diseases. As early as 1953, interest in gastro-intestinal inflammation in psychosis was raised, when a group of researchers found in an autopsy study that out of 82 patients with schizophrenia, 50% had gastritis, 88% enteritis and 92% colitis (92). This study has not since been replicated, but other signs of microbiome dysbiosis in this group of patients has been found with significant difference between cases and controls in the presence of both bacteria and fungi (93), and bacteriophages (94). Studies so far have mainly focused on the oropharyngeal microbiome due to practical limitations. One study however, has looked into fecal microbiome, finding no significant difference between healthy controls and patients, but showing associations between microbiome composition and symptom severity and outcome (95).

The composition of the microbiome has been hypothesized to be very important in the development of both the central nervous system (96) and the immune system (97, 98). Dysbiosis of the microbiome has been shown to affect both the Th1/Th2 balance and the ratio of T regulatory and Th17 cells, impacting the immune response to foreign pathogens (99). Dysbiosis have been found to influence the T-cell mediated inflammation in MS patients (100, 101), and has also been suspected to play a part in the development of celiac disease (102), as well as non-gastro-intestinal autoimmune diseases (103). In rodents, disruption of the microbiome has been found to impair social functioning (104), behavior and cognition (105), and induce neurodevelopmental disorders (106).

An important function of the microbiome, seems to be its effect on the epithelial cells in the GI wall, with evidence implicating that the composition of the microbiome is important for the tightness of the gut-blood barrier (107). Severance et al. (108) found markers in the serum of patients with schizophrenia indicating increased permeability, also known as “leaky gut.” A leaky gut allows the entrance of foreign pathogens and antigens into the blood. It has been suspected to induce systemic inflammation, and in mice it has been found to even result in neuroinflammation (109), both of which might increase the risk of mental illness and autoimmune diseases.

Interestingly, both infections and the treatment hereof with antibiotics can modulate the microbiome, linking the previously mentioned epidemiological findings of the influence of infections (26) with the microbiome theory. Additionally, it has been theorized that maternal infection might alter both the maternal and fetal microbiome (110), possibly impacting the immune system and neuropsychiatric development of the offspring.

A few studies have tried probiotic treatment in patients with schizophrenia, but no evidence of effect hereof on psychopathology has yet been found (111, 112). However, further research on the actual composition of the microbiome in patients with mental illness as well as the possibility of using probiotics as treatment hereof is warranted.

### Psychological Stress

Psychological stress such as sexual abuse, physical abuse, emotional/psychological abuse, neglect, parental death, and bullying, both in childhood and later on, has been associated with increased risk of psychotic disorders in multiple studies (113, 114). A Swedish register-based study found that stress-related disorders increased the risk of subsequent development of autoimmune disorders (115) and, accordingly, in many other studies, stress have been found to be associated with disease onset and disease exacerbations in several autoimmune conditions (116).

Stress can theoretically influence many of the above-mentioned possible etiological factors. Acute psychological stress, even in brief episodes, have been found in a meta-analysis to increase circulating proinflammatory cytokines such as IL-6, IL-1β, and TNF-α (117), possibly via the sympathetic nerve system and the HPA axis, and multiple adverse life events or stressful living conditions might therefore possibly contribute to a more chronic inflammatory state with dysregulation of immune response (118).

Psychological stress have been thought to influence composition of the microbiome and vice versa, as well as the microbiome's effect on peripheral inflammation (119). Additionally, it has been hypothesized to increase susceptibility to infections, with one study finding that healthy subjects with higher scores on questionnaires on psychological stress were more prone to developing clinical cold and respiratory infections after exposure to respiratory viruses (120). Acute stress, for example as a result of a psychiatric disorder or hospitalization, may also lead to exacerbation in symptoms of autoimmune diseases, leading to the discovery of a disease formerly undiagnosed.

## Clinical Implications

The increasing knowledge on the potential involvement of inflammatory processes in mental disorders and the associations found between autoimmunity and psychotic disorders can help the expanding field of immuno-psychiatry and have impact on the outcome of patients. In the last couple of years, researchers have focused on the role of infections, autoantibodies and other immune components that plays a major role in autoimmune diseases. Potentially this might also be the case for mental disorders. Risk factors for both autoimmune diseases and schizophrenia includes an interaction between environmental factors, such as infections and stress, with genetic factors involving the HLA region. Autoimmune reactions with activation of immune components and the production of NSAbs can induce a broad spectrum of psychiatric symptoms, hereunder psychosis. The potential autoimmune-mediated psychosis group might only be a small part of a broader immune-related psychosis group, and an even smaller fraction of the overall psychosis group. However, identification of this subgroup might allow for precision medicine strategies where immune-based treatment could possibly improve the psychotic symptoms. A quick discovery and treatment of autoimmune encephalitis markedly reduces the neuropsychiatric sequelae, and intensive immunotherapy in lupus patients with psychosis massively benefits psychiatric symptoms (42, 121).

Focus on the association between autoimmunity and psychosis, regardless of etiology, is important, not only for researchers but also for the individual patient. It is known that patients suffering from schizophrenia have an excess early mortality, with a life expectancy up to 13.5 years shorter than the general population, primarily due to physical diseases (122). Bearing this in mind and considering that patients with psychotic disorders might struggle with reporting on somatic symptoms, it is important for clinicians to be aware of an increased prevalence of autoimmune disease in this group. Symptoms from a disease such as celiac disease or rheumatoid arthritis might very well be overlooked and cast aside as a part of the patient's psychosis, or possibly adverse events caused by their treatment. With increasingly sufficient treatment strategies in autoimmune diseases, overlooking and therefore not treating these diseases, increases the health gap between those with schizophrenia and the general population even further. Therefore, patients with a psychotic disorder need to be thoroughly and frequently examined when presenting with symptoms possibly related to autoimmunity or other health problems.

## Author Contributions

RJ was responsible for literature search and wrote the first draft of the manuscript. MB contributed with supervision and expert advice and revised the manuscript. All authors contributed to manuscript revision, read and approved the submitted version.

### Conflict of Interest Statement

The authors declare that the research was conducted in the absence of any commercial or financial relationships that could be construed as a potential conflict of interest.
